# Post-Exercise Recovery Following 30-Day Supplementation of Trans-Resveratrol and Polyphenol-Enriched Extracts

**DOI:** 10.3390/sports7100226

**Published:** 2019-10-20

**Authors:** Edward Jo, Rachel Bartosh, Alexandra T. Auslander, Dean Directo, Adam Osmond, Michael WH Wong

**Affiliations:** Human Performance Research Laboratory, Department of Kinesiology and Health Promotion, California State University Pomona, Pomona, CA 92805, USAataylor@cpp.edu (A.T.A.); dean.directo@gmail.com (D.D.); adosmond7@gmail.com (A.O.); mike.wong203@gmail.com (M.W.W.)

**Keywords:** polyphenols, supplementation, skeletal muscle

## Abstract

Background: The purpose of this study was to investigate the effects of 30-day consumption of trans-resveratrol and polyphenol-enriched extracts on indices of exercise-induced muscle damage (EIMD) and performance following eccentric-loaded resistance exercise (ECRE). Methods: Following 30 days of resveratrol-polyphenol (RES) (*n* = 10) or placebo control (CTL) (*n* = 12) supplementation, subjects performed a bout of ECRE to induce EIMD. EIMD biomarkers, perceived soreness, pain threshold and tolerance, range of motion, and performance were measured before and 24 and 48 h after ECRE. Results: CTL subjects demonstrated increased soreness at 24 (*p* = 0.02) and 48 h (*p* = 0.03) post-ECRE, while RES subjects reported increased soreness at 24 h post-ECRE (*p* = 0.0003). CTL subjects exhibited decreased pain threshold in the vastus lateralis at 24 h post-ECRE (*p* = 0.03). CTL subjects also displayed decreased pain tolerance in the vastus intermedius at 24 h post-ECRE (*p* = 0.03) and the vastus lateralis at 24 (*p* = 0.003) and 48 h (*p* = 0.003). RES participants showed no change in pain threshold or tolerance from baseline. CTL subjects showed a decrease in mean (*p* = 0.04) and peak power (*p* = 0.04) at 24 h post-ECRE, while RES participants demonstrated no changes from baseline. No between-group differences were observed for the changes in serum creatine kinase. Serum C-reactive protein increased similarly in both groups at 24 h post-ECRE (*p* < 0.002), remaining elevated in CTL subjects while RES participants demonstrated a decline from 24 to 48 h (*p* = 0.04). Serum interleukin 6 increased at 24 h post-ECRE in both groups (*p* < 0.003) followed by a decrease from 24 to 48 h, returning to baseline levels only for RES subjects. Conclusion: Trans-resveratrol and polyphenol-enriched extract supplementation may support the attenuation of soreness and inflammation and improve performance recovery following ECRE without modulation of indirect biomarkers of EIMD.

## 1. Introduction

Skeletal muscle may undergo cellular level damage in response to unaccustomed exercise especially when eccentric contractions are stressed within the movements. This is commonly referred to as exercise-induced muscle damage (EIMD) which often manifests in transient performance decline, soreness and swelling, and release of intramyocellular proteins into circulation, all of which are common indirect markers of EIMD. Increased reactive oxygen species (ROS), disruption of muscle fiber structures and integrity, acute-phase immune response, and inflammation have all been purported mechanisms underlying EIMD and its related symptoms like localized hyperalgesia [1]. Consequently, EIMD may significantly limit performance during subsequent exercise bouts, athletic competition, or occupational tasks. Therefore, a variety of prophylactic and therapeutic strategies have been previously researched and employed in practice to mitigate the acute negative effects of EIMD on performance. One of the most commonly used strategies include nutritional intervention, which often incorporates dietary supplementation [2]. Although amino acid-based supplements like essential amino acids and complete intact protein sources are considered traditional approaches, nutrients with antioxidant and/or anti-inflammatory properties have been widely purported as a countermeasure strategy for EIMD and related symptoms.

The recent growth of interest in antioxidant and anti-inflammatory nutrients in aiding short-term recovery from EIMD has largely been enabled by emerging evidence demonstrating the interaction among oxidative stress, inflammation, and EIMD symptoms, including soreness and temporary performance decline [3,4]. Among these, polyphenols are bioactive compounds abundantly found in various types of fruits and vegetables that possess the ability to modulate inflammation directly or indirectly via oxidative stress mitigation. In brief, exogenous polyphenols have shown to convert ROS produced during the acute response to cellular damage into less reactive molecules or suppress less reactive ROS. Previous evidence demonstrates the potential benefits of polyphenol consumption in the context of EIMD and recovery as long-term supplementation has shown efficacy in preserving muscular strength following intense eccentric-exercise [5] and reducing muscle protein degradation [6], soreness [7], and ROS levels [8]. Also, short-term consumption of a polyphenol-infused beverage containing black grape, raspberry, and red currant, prior to, during and immediately following a cycle ergometer test reduced the levels of plasma creatine kinase and oxidative stress [6]. Furthermore, previous evidence showed preservation of isometric muscular force following eccentric exercise preceded by a 15-day consumption of pomegranate juice, an abundant source of polyphenols, although no attenuation of soreness was observed [5]. Polyphenol compounds found in grapes, including catechins, resveratrol, and quercetin, have also been known to exhibit antioxidant and anti-inflammatory properties [9]. For instance, purple grape juice was able to counteract oxidative damage in the brain, skeletal, and blood tissues of rats induced by muscle-damaging exercise [10]. Many sources for polyphenols, including quercetin [11], green tea [12], and pomegranate juice [5], have been studied in regard to their antioxidant effects and have shown promising results. Among the variety of polyphenols, resveratrol has recently garnered an increasing level of attention due to emerging empirical evidence demonstrating its potential benefits for exercise recovery.

Resveratrol is a naturally occurring polyphenol produced in plants and insects in response to biological stresses such as parasites, fungal infections, ultra-violet radiation, and various chemical substances such as pesticides [13,14]. It is found most abundantly in blueberries, blackberries, peanuts, and red wine, with the richest source being grape skin [13]. Resveratrol has previously demonstrated anti-cancer, antioxidant, and anti-inflammatory effects as well as metabolic and cardiovascular support [15]. Resveratrol has shown some potency for scavenging free radicals produced by oxidative stress due to its antioxidant properties [8]. Moreover, previous findings support recent views of resveratrol as a pseudo exercise mimetic due to evidence, albeit limited, indicating improved bioenergetic capacities and exercise performance following long-term supplementation [16]. Udani et al. [7] reported efficacy of a proprietary resveratrol-based dietary supplement in attenuating post-exercise soreness after 30 days of intake, although biomarkers of EIMD were not significantly modulated [7]. Based on the aforementioned studies, there are some evidence that support the use of resveratrol and polyphenol supplementation as a strategy to reduce soreness, however its effects on performance recovery following EIMD has yet been elucidated. Moreover, there is a lack of applicable data that can properly guide the practical application of resveratrol-based supplementation in human performance and recovery. 

Therefore, supplementation of polyphenols, like resveratrol, could potentially minimize the effects of EIMD and aid in the functional recovery from damaging exercise. A controlled trial examining the effects of long-term polyphenol supplementation on markers of muscle damage following eccentric-based exercise would improve the current knowledge regarding the application and usefulness of dietary polyphenols as a strategy to minimize EIMD and/or its symptoms. The purpose of this study was to investigate the effects of supplementation of *trans*-resveratrol and polyphenol-enriched extracts on (1) functional, biochemical, and perception-based indices of EIMD and (2) performance recovery following high intensity, eccentric-based exercise in resistance-trained males and females. 

## 2. Materials and Methods

### 2.1. Experimental Design

**Figure 1:**
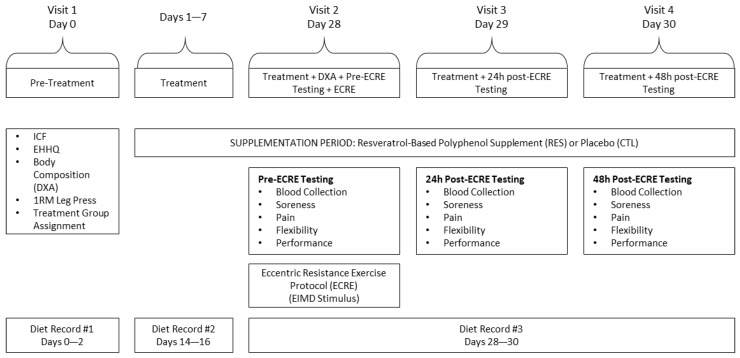
Schematic of the experimental timeline. ICF = informed consent form, EHHQ = Exercise Health History Questionnaire, DXA = dual-energy X-ray absorptiometry, 1RM = one-repetition maximum, ECRE = eccentric resistance exercise, EIMD = exercise-induced muscle damage.

A randomized, double blind, placebo-controlled, between-group design was implemented for the current study (Figure 1). Participants visited the Human Performance Research Laboratory at California State Polytechnic University, Pomona on four separate occasions over 30 days. During the first laboratory visit, participants underwent assessments for exercise and health history, body composition via dual energy X-ray absorptiometry (DXA), and one-repetition maximum on the leg press. 

During the first visit, participants were randomly allocated to one of the following groups: (1) the resveratrol-based polyphenol supplement (RES) group (6 females, 4 males), and the placebo control (CTL) group (6 females, 6 males). The RES and CTL groups underwent 30 days of resveratrol-based polyphenol and placebo supplementation, respectively. 

Four weeks following the first visit (on day 28), participants returned to the laboratory for the post-treatment DXA scan. Following blood collection, participants underwent baseline assessments for soreness, pain threshold and tolerance, flexibility, and muscular performance in the listed order. Participants then performed an eccentric resistance exercise (ECRE) protocol to induce EIMD (described below). Participants repeated the aforesaid assessments (minus DXA) 24 and 48 h following the ECRE. During these testing periods, participants continued to consume their assigned supplement. Each participant underwent protocols administered by the same investigators for all laboratory visits. 

### 2.2. Participants

Twenty-two healthy college-aged, resistance-trained males (*n* = 10) and females (*n* = 12) participated in this study. A total of 24 subjects were recruited; however, two male participants were dismissed from the study due to reasons pertaining to adherence. Each volunteer initially completed a pre-participation exercise and healthy history questionnaire as well as signed an informed consent form. Subjects were required to meet the following inclusion criteria to participate in this research study: (1) age = 18 to 32 years, (2) not a competitive athlete in a collegiate or professional sport, and (3) recreationally resistance-trained as defined by resistance exercise performed 3–4 days/week for 6 months prior to the start of the study and able to perform at least one repetition on the leg press with a load 1.5 (male) or 1.0 (female) times their bodyweight. Subjects were excluded from participation if they reported or exhibited: (1) history of medical or surgical events in which the study protocols would be contraindicated or confound the interpretation of the results. These included, but were not limited to, cardiovascular, metabolic, pulmonary, renal, or kidney diseases, hypertension, or musculoskeletal impediments; (2) use of any medication including those with cardiovascular, pulmonary, thyroid, hyperlipidemic, hypoglycemic, hypertensive, endocrinological, psychotropic, neuromuscular, neurological, or androgenic implications; (3) pregnancy; (4) daily use of ergogenic aids or dietary sports supplements within 6 weeks prior to the study (use of nutritive supplements, e.g., whey protein, was permissible); (5) daily intake of foods containing high amounts of resveratrol including grapes, blueberries, cranberries, pomegranates or pomegranate juice, peanuts, flaxseed, linseed, chia seeds, or red or white wine; or (6) daily use of anti-inflammatories, aspirin, pain relievers, non-steroidal anti-inflammatory drugs (NSAIDS), muscle relaxers, or fish oil (omega 3/omega 6). Participants were instructed to maintain their habitual physical activity/exercise during participation which was monitored weekly via questionnaire. However, during week 4 of supplementation, subjects were asked to stop physical activity/exercise outside of the study. All subjects signed an informed consent form prior to participation, and this study was approved by the Institutional Review Board at California State Polytechnic University, Pomona (#IRB-16-173). 

### 2.3. Eccentric Resistance Exercise Protocol (EIMD Stimulus)

Participants completed five sets of 20 vertical depth jumps with 10 s of rest between each repetition and a 2-min rest period between each set. Prior investigations have demonstrated that the performance of depth jumps sufficiently induced EIMD [17]. Vertical depth jumps were performed from a platform 24 inches (61 cm) tall. Upon landing, participants were encouraged to immediately jump vertically with maximal effort. Following the depth jumps, participants performed 2 sets of 6 repetitions of the leg press exercise with a load that was 90% of their one-repetition maximum (1RM). The eccentric portion of the exercise was performed at a rate of 3 s per contraction aided by a metronome set at 60 beats per minute. The concentric portion was performed with maximum effort with assistance from the investigator if needed.

### 2.4. Dietary Supplementation

In a double-blind manner, the RES and CTL groups consumed one serving per day of a resveratrol-based polyphenol supplement or placebo, respectively for 30 days (Shaklee, Pleasanton, CA, USA). The 30-day period was determined based on a prior investigation of a resveratrol supplement [7]. The RES supplement was a 716 mg/serving concentrate blend of muscadine grape extract, trans-resveratrol (standardized to minimum 98% purity), red wine extract, pomegranate extract, chebulic myrobalan extract, black currant extract, purple carrot extract, medium-chain triglyceride oil, gelatin, glycerin, yellow beeswax, soy lecithin, caramel color, and red grape juice extract. Total resveratrol content was 60 mg/serving. The placebo was isocaloric and taste- and appearance-matched. All supplements were in white capsule form. Non-transparent bottles containing each supplement were labeled either “A” or “B”, and information regarding the content of the bottles were sealed in an envelope until the completion of data analysis. 

### 2.5. Body Composition

Body composition was measured by dual-energy x-ray absorptiometry (DXA) (Hologic Discovery-QDR Series Densitometer, Bedford, MA, USA). The DXA machine was calibrated before each scan using a manufacturer-provided phantom. All DXA measurements and analyses were conducted by a single certified technologist.

### 2.6. Muscular Strength

A one-repetition maximum (1RM) assessment was used to assess participants’ muscular strength for the leg press exercise. The protocol employed is explained elsewhere [18]. A 1RM was defined as the greatest load (i.e., weight) that can be properly moved through the entire range of motion for a given exercise for no more than one complete repetition. Data were used only for load determination for the ECRE.

### 2.7. Muscle Soreness

Soreness was assessed using the visual analogue scale (VAS) method [5]. The scale consisted of a line measuring from 0 cm to 10 cm, with a rating of 0 cm indicating “no pain”, 5 cm indicating “moderate pain”, and 10 cm indicating “unbearable pain”. The VAS technique has been employed by prior investigations and can reproduce 90% of perceived ratings within 9 nm. Participants were instructed to indicate perceived soreness level on the VAS while sitting with a relaxed lower body representing resting muscle soreness. The subjects then performed and held a squat at a 90° knee angle for 3 s whilst rating their perceived soreness level on the VAS. This represented perceived soreness under muscular tension. 

### 2.8. Localized Pain Threshold and Tolerance

An algometer was utilized to identify the pressure and/or force eliciting a pressure pain threshold and pain tolerance [19]. The pain threshold was defined as the minimum pressure/force that induces pain in tender tissue while the pain tolerance was the point where an applied pressure stimulus could no longer be tolerated. The algometer test was administered on the mid- vastus lateralis, vastus intermedius, and gastrocnemius muscles of both limbs and averaged for data analysis. For pain threshold assessments, the flat-end tip of the algometer was applied with gradually increasing force until the subject verbally indicated the presence of pain. For pain tolerance measurements, the tip of the algometer was applied with gradually increasing force until the subject verbally indicated that the pain could no longer be tolerated.

### 2.9. Lower-Body Flexibility

A stand-and-reach test was employed in which subjects were instructed to stand barefoot on top of a 12-inch platform with the top of the platform being the relative zero point [17]. Subjects were asked to slightly bend their knees and reach down as far as possible with straight arms until they could not proceed any further. Subjects were to hold this position for measurement to be taken by a measuring tape. All points above relative zero indicated a negative measurement whereas all points below relative zero indicated a positive measurement. The score was recorded to the nearest cm. The subject was provided a single trial to prevent any carry-over effect from previous attempts. 

### 2.10. Lower-Body Dynamic Power and Fatigue

Participants were asked to complete one set of a maximum of 20 repetitions (i.e., perform as many repetitions as possible while using proper technique) on the leg press exercise using a load that was 60% of their predetermined 1RM. Subjects were instructed to perform the concentric portion of the repetition with maximum effort and velocity while the eccentric portion was controlled at a rate of 3 s. The total number of repetitions performed were counted, whilst repetitions not meeting the criteria for proper technique were rejected, inherently by the linear position transducer (TENDO Sports Machines, Trencin, Slovak Republic). The device encloses a linear transducer that was attached next to the seat of the leg press. The line was tethered to the leg press platform. This device consequently measured the linear displacement of the platform through each repetition and the time required to perform the repetition, thereby calculating velocity, peak power, and mean power. 

### 2.11. Biomarkers of Muscle Damage and Inflammation

Two 200-microliter samples of capillary blood were collected via sterile finger-prick and capillary blood collection techniques. Blood samples were allowed to clot for 30 min at room temperature and then centrifuged for 10 min for serum separation. All serum samples were aliquoted and stored at −20 °C until analysis. Serum creatine kinase (CK), C-reactive protein (CRP), and interleukin-6 (IL6) (Abcam, Cambridge, UK) were analyzed using enzyme-linked immunoassay Absorbance was measured via microplate reader (ELx800, Biotek, Winooski, VT, USA) using assay-specific wavelengths. All samples were analyzed in duplicates (coefficient of variation = 2.3%).

### 2.12. Control for Diet

Subjects provided three 3-day diet records that included the type and amount of foods and beverages consumed by the subject during the three days. The first diet record was provided by the subject one day prior to supplementation as well as the first two days of supplementation on days 0–2 (Figure 1). The second diet record was provided during the second week of the 4-week supplementation period on days 14–16. The third diet record was provided on Visit 2 and the two days following on days 28–30 (visits 3 and 4). The subjects were notified and reminded one day prior to the day the diet record was to be provided. All diet records were analyzed using The Food Processor^®^ Nutrition and Fitness Software (ESHA Research, Salem, OR, USA).

### 2.13. Analysis of Data

A 2 (group) × 3 (time) × 2 (sex) repeated measures analysis of variance (ANOVA) was used to detect main effects and interactions and a post-hoc test with Bonferroni adjustment was used for pairwise comparisons. In addition, mean power (MP) and peak power (PP) at 24 and 48 h post-ECRE were converted to a percent-of-baseline score; pain threshold measures at each post-exercise time point were converted to a change (∆) from baseline value. Subsequently, between-group comparisons at each post-ECRE time point for these data were performed using a one-way analysis of covariance (ANCOVA) with baseline measures as a covariate. In the event of a significant F-ratio, a Tukey’s post hoc test was used for pairwise comparisons. Also, between-group comparisons for area under the curve (AUC) for biomarkers were analyzed using an independent *t*-test. All statistical analyses were performed using the Statistical Package for Social Science (SPSS 23, IBM Corporation, Armonk, NY) with significance set at *p* < 0.05. All data presented as mean ± SD.

## 3. Results

### 3.1. Descriptive and Control Variables

There were no significant differences between groups for any baseline descriptive measures (Table 1). Also, no significant between group differences were detected for mean caloric (*p* = 0.67), carbohydrate (*p* = 0.92), total fat (*p* = 0.60), saturated fat (*p* = 0.20), polyunsaturated fatty acid (*p* = 0.56), monounsaturated fatty acid (*p* = 0.67), or protein (*p* = 0.94) intake during the study period. There were no significant changes in body composition measurements between day 0 and day 28 (after the supplementation period).

### 3.2. Muscle Soreness Perception

#### 3.2.1. Resting Soreness

**Figure 2:**
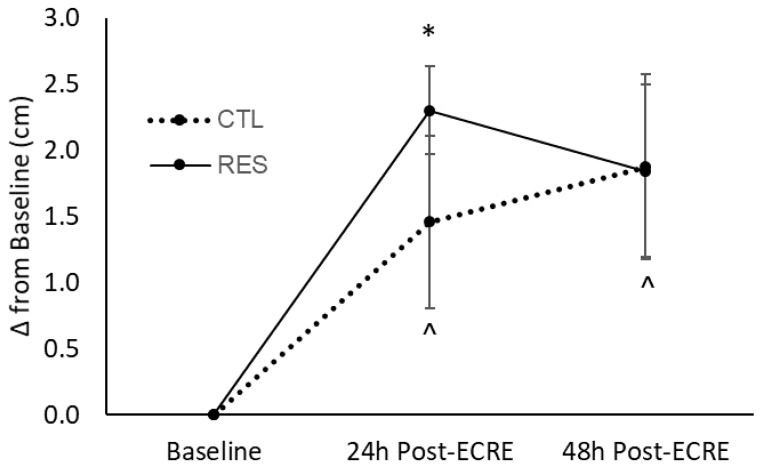
Perceived muscle soreness at rest at baseline, 24- and 48-h post-ECRE. ECRE = eccentric resistance exercise, CTL = placebo, RES = resveratrol. * Significant increase from baseline (*p* = 0.0003); ^ Significant increase from baseline (*p* < 0.05).

There was a main effect for time for resting soreness (i.e., not under muscular tension) (*p* = 0.0001) (Figure 2). Post-hoc test revealed increased soreness at 24- (*p* = 0.02, +1.5 cm, 95%CI = −2.7, −0.2) and 48-h (*p* = 0.03, +1.9 cm, 95%CI = −3.6, −0.2) post-ECRE compared to baseline for the CTL group. The RES group showed increased soreness from baseline to 24 h post-ECRE (*p* = 0.0003, +2.5 cm, 95%CI = −3.8, −1.2); soreness at 48 h was not different from baseline. There was no effect of sex for resting soreness measurements. 

#### 3.2.2. Soreness under Muscular Tension

**Figure 3:**
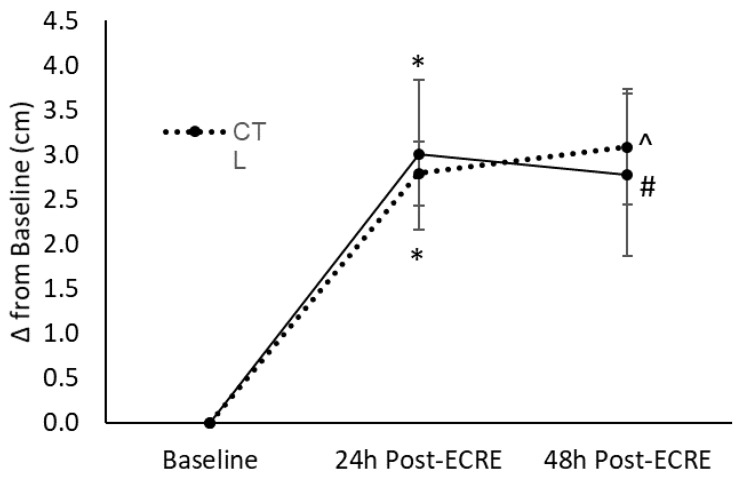
Perceived muscle soreness under muscular tension at baseline, 24- and 48-h post- ECRE. ECRE = eccentric resistance exercise, CTL = placebo, RES = resveratrol. * Significant increase from baseline (*p* < 0.0005); ^ Significant increase from baseline (*p* = 0.01); # Significant increase than baseline (*p* = 0.007)

There was a main effect for time for soreness under muscular tension (*p* = 0.0001) (Figure 3). The CTL group demonstrated increased soreness at 24- (*p* < 0.0005, +2.8 cm, 95%CI = −4.3, −1.3) and 48-h (*p* = 0.01, +3.1 cm, 95%CI = −5.0, −1.2) post-ECRE compared to baseline. The RES group also showed increased soreness from baseline to 24- (*p* < 0.0005, +3.2 cm, 95%CI = −4.9, −1.5) and 48-h (*p* = 0.007, +2.8 cm, 95%CI = −4.9, −0.7) post-ECRE. There was no effect of sex for soreness under muscular tension. 

### 3.3. Localized Pain Threshold and Pain Tolerance

#### 3.3.1. Pain Threshold

Results for pain threshold are displayed in Table 2. The CTL group exhibited a decreased pain threshold in the vastus lateralis from baseline to 24 h post-ECRE (*p* = 0.03, −15.8 N). There was no effect of sex for pain threshold.

#### 3.3.2. Pain Tolerance

Results for pain tolerance are displayed in Table 3. The CTL group exhibited decreased pain tolerance in the vastus intermedius from baseline to 48 h post-ECRE (*p* = 0.03, −15.9 N). The CTL group also demonstrated decreased pain tolerance in the vastus lateralis from baseline to 24- (*p* = 0.003, −33.7 N) and 48-h post-ECRE (*p* = 0.003, −36.7 N). There was no effect of sex for pain tolerance.

### 3.4. Lower-Body Flexibility

**Figure 4:**
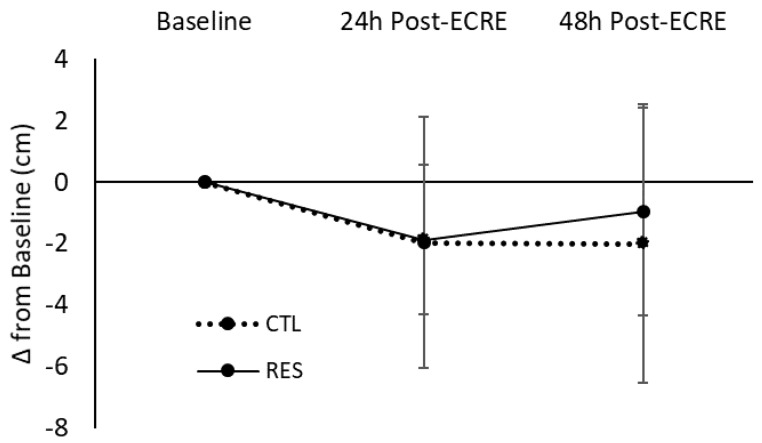
Change in lower-body flexibility at 24- and 48-h post-ECRE. ECRE = eccentric resistance exercise, CTL = placebo, RES = resveratrol.

There was a main effect for time for lower-body flexibility (*p* = 0.03); however, there were no significant group by time interactions detected (Figure 4). There was no effect of sex for lower-body flexibility.

### 3.5. Lower-Body Dynamic Power

**Figure 5:**
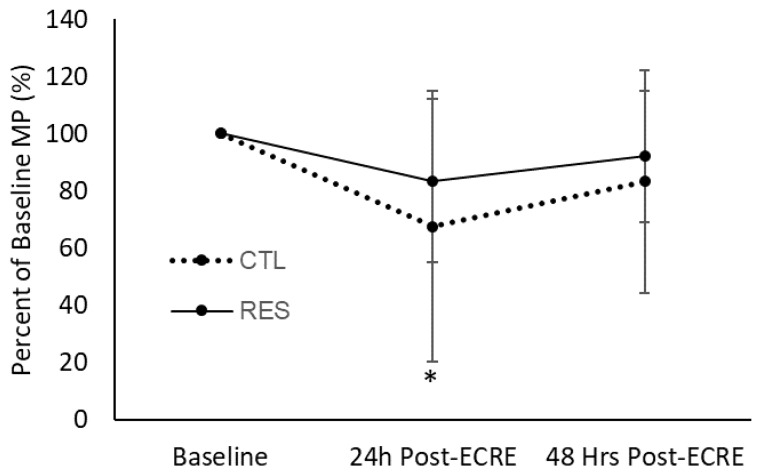
Mean power as a percentage of baseline at 24- and 48-h post-ECRE. MP = mean power, ECRE = eccentric resistance exercise, CTL = placebo, RES = resveratrol. * Significant decrease from baseline (*p* = 0.04).

**Figure 6:**
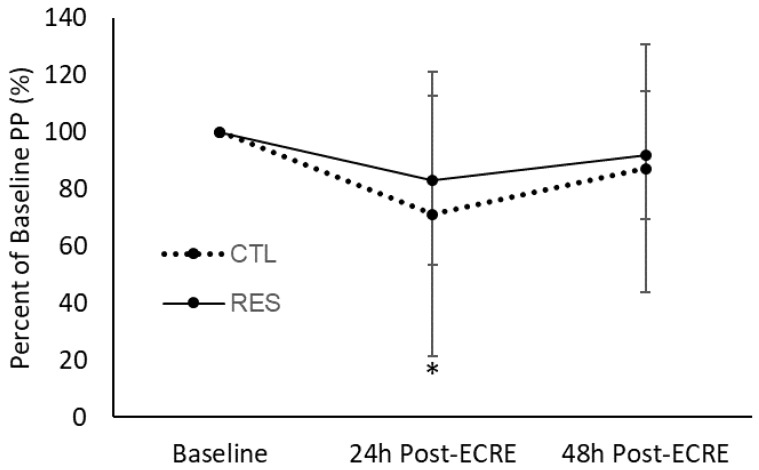
Peak power as a percentage of baseline at 24- and 48-h post-ECRE. PP = peak power, ECRE = eccentric resistance exercise, CTL = placebo, RES = resveratrol. * Significant decrease from baseline (*p* = 0.04).

The CTL group demonstrated a decrease in lower body dynamic mean power (MP) (*p* = 0.04, 67.6% of baseline) and peak power (PP) (*p* = 0.04, 71.1% of baseline) from baseline to 24 h post-ECRE (Figure 5 and Figure 6). MP or PP at 48 h post-ECRE did not differ from baseline for the CTL group. The RES group exhibited no significant changes in MP or PP at any post-ECRE timepoints from baseline. There was no effect of sex for MP or PP. 

### 3.6. Muscle Damage and Acute Inflammation Biomarkers

**Figure 7:**
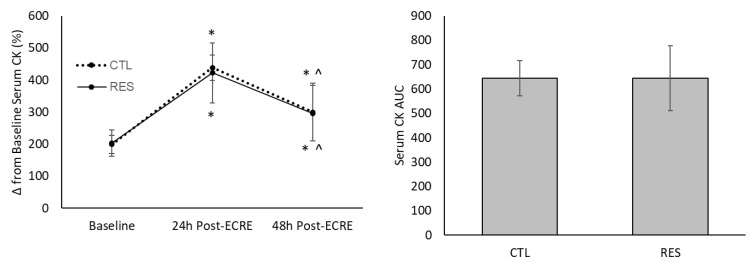
Serum creatine kinase (CK) at baseline and 24- and 48-h post-ECRE (left), and area under the curve (AUC) (right). ECRE = eccentric resistance exercise, CTL = placebo, RES = resveratrol. * Significant increase from baseline (*p* < 0.005); ^ Significant decrease from 24 h post-ECRE (*p* < 0.007).

**Figure 8:**
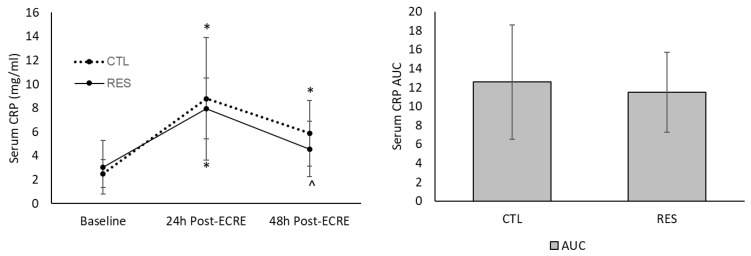
Serum C-reactive protein (CRP) at baseline and 24- and 48-h post-ECRE (left), and area under the curve (AUC) (right). ECRE = eccentric resistance exercise, CTL = placebo, RES = resveratrol. * Significant increase from baseline (*p* < 0.002); ^ Significant decrease from 24 h post-ECRE (*p* = 0.04).

**Figure 9:**
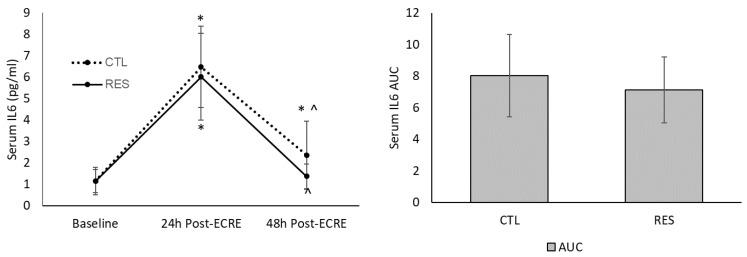
Serum interleukin 6 (IL6) at baseline and 24- and 48-h post-ECRE (left), and area under the curve (AUC) (right). ECRE = eccentric resistance exercise, CTL = placebo, RES = resveratrol. * Significant increase from baseline (*p* < 0.003); ^ Significant decrease from 24 h post-ECRE (*p* < 0.0001).

There was a main time effect for serum CK, CRP, and IL6 levels (*p* < 0.0001) (Figure 7, Figure 8 and Figure 9). Both groups demonstrated a similar increase (*p* < 0.001) in CK from baseline to 24 h post-ECRE (CTL group = +124.9%; RES group = +110.3%) and 48 h post-ECRE (CTL group = +48.8%; RES group = +47.3%). There was also a similar decrease in CK between groups from 24–48 h post-ECRE (*p* < 0.006) (CTL group = –30.6%; RES group = –26.3%). There was no between-group difference for CK area under the curve (AUC).

CRP levels increased similarly between groups from baseline to 24 h post-ECRE (*p* <0.001) (CTL group = +268.3%; RES group = +251.7%) (Figure 8). Only the CTL group demonstrated an increase in CRP from baseline to 48 h post-ECRE (+205.8%; *p* = 0.001). Only the RES group showed a decrease in CRP from 24–48 h post-ECRE (–40.4%; *p* = 0.04). There was no between-group difference for CRP AUC.

IL6 levels increased similarly between groups from baseline to 24 h post-ECRE (*p* < 0.001) (CTL group = +642.5%; RES group = +651.8%) (Figure 9). Only the CTL group demonstrated an increase in CRP from baseline to 48 h post-ECRE (+183.8%; p = 0.002). Both groups showed a decrease in CRP from 24–48 h post-ECRE (*p* < 0.003) (CTL group = –63.0%; RES group = 74.2%). There was no between-group difference for IL6 AUC. No effect of sex was detected for any of the biomarkers. 

## 4. Discussion

The overall objective of this study was to investigate the acute effects of a 30-day supplementation of trans-resveratrol and polyphenol-enriched extracts containing 60 mg of resveratrol per serving on muscle damage, acute inflammation, hyperalgesia, and performance following a bout of ECRE. As an executive summary of the principal findings, 30 days of polyphenol/resveratrol supplementation showed slight efficacy in the preservation of performance during a state of EIMD (indirectly marked by elevated CK levels). These performance outcomes were not accompanied, however, by attenuation of CK leakage compared to CTL, although localized hyperalgesia and delayed onset muscle soreness (DOMS) were lessened. Thus, multi-day consumption of the current experimental supplement appears to lack potency for the reduction of EIMD although its symptoms were slightly mitigated. It must be noted, however, a comparison of serum CK levels may be inadequate to infer small differences in the degree of muscle damage. Regardless, within the limits of the current study it appears that decreased muscle damage cannot explain the acute preservation of muscular power by the RES group following ECRE. With that said, further questions emerge regarding the role of resveratrol in modulating post-exercise oxidative stress, a stimulus of secondary damage during the acute inflammatory response to EIMD. In theory, resveratrol, as a known antioxidant [20], would conceivably reduce muscle damage (specifically secondary damage) through free radical neutralization and attenuation of oxidative stress. Unfortunately, the current investigation lacked measures of oxidative stress biomarkers and antioxidant capacity, e.g., Trolox equivalent antioxidant capacity [21], due to errors during analysis. However, the available data allude to potential anti-inflammatory effects of multi-day resveratrol supplementation. According to a recent review and meta-analysis of 15 randomized clinical trials, resveratrol appears to effectively reduce serum CRP levels while other prominent inflammatory markers such as tumor necrosis factor alpha and IL6 remain relatively unaltered [22]. It must be emphasized these outcomes were mostly observed in clinical cases of chronic inflammation. Despite no group differences in inflammatory biomarkers at any post-ECRE timepoint, CRP and IL6 returned to baseline levels by 48 h post-ECRE in resveratrol-supplemented subjects whilst remaining elevated above baseline levels in the placebo group. 

Prior research has characterized the innate inflammatory response to EIMD in humans and has described its essential role in the adaptive processes of skeletal muscle during chronic high-force and high-tension exercise [4,23]. Moreover, acute inflammation induced by EIMD contributes to the common hyperalgesia and soreness experienced after heavy exercise via modulation of noxious pathways, a process complementary to healing but transitorily counteractive to performance. Thus, acute inflammation has become a biological target for recovery strategies between sporting events or exercise, particularly when training adaptation is inconsequential (e.g., in-season competition). This may contribute to the emerging use of anti-inflammatory drugs, e.g., non-steroidal anti-inflammatory drugs (NSAIDs), by athletes for the sake of facilitating performance recovery (not chronic adaptation per se). Although our data concerning post-ECRE inflammation is short of compelling evidence, it may be suggestive of the benefits of resveratrol as a non-pharmacological, and thus, safer approach to managing DOMS and promoting recovery performance (not necessarily repair of muscle fibers). In fact, evidence from mechanistic animal studies demonstrate parallels between resveratrol and NSAIDs as an inhibitor of cyclooxygenase-2 (COX2) and prostaglandin E2 (PGE2), which are prominent markers of a pathway linking inflammation and pain, especially during tissue perturbations [24,25,26]. Although little inferences to post-exercise recovery scenarios can be made by the current biomarker data, they may provide at least partial explanation to the observed effects of resveratrol on soreness and pain sensitivity.

Although animal studies have shown positive performance and recovery outcomes associated with resveratrol administration, the limited body of evidence on human subjects has been mixed. For instance, O’Connor et al. (2013) contrastingly found that a twice daily supplementation of a resveratrol-containing grape beverage consumed for 42 days failed to mitigate the strength reductions and soreness following heavy eccentric exercise versus placebo [9]. These differential results suggest that various factors influence the efficacy by which resveratrol and/or polyphenols can mitigate performance declines associated with EIMD, such as food source and dosage. The grape beverage used by O’Connor et al. (2013) consisted of 17.5 mg/serving of a resveratrol-polyphenol blend whereas the supplement in the current study contained 716 mg/serving of polyphenol sources with 60 mg/serving of *trans*-resveratrol. These differential outcomes lead to the question of whether resveratrol-induced effects on EIMD-induced soreness is dose-dependent. Previous studies such as Wu et al. (2013) suggest that perhaps greater doses of resveratrol are more beneficial in terms of performance and recovery. The same study investigated a dosing spectrum of 0–125 mg/kg of body mass/day and found that there was a dose-dependent effect with the higher amount demonstrating greater benefits [27]. Thus, from a practical perspective, higher absolute or relative (to body mass) doses of resveratrol may be more ideal for recovery support, although there is limited knowledge of proper application in human models. Overall, a relatively small quantity of research has explored the application of resveratrol supplementation on recovery of muscular function and/or performance following a damaging exercise bout. Although discrepancies exist between the outcomes of the present study and those by O’Connor et al. [9], the current results demonstrate the potential of resveratrol supplementation as an effective aid for performance recovery. Indeed, the facilitation of performance recovery rate would be conceivably influenced by several interlinked factors such as, intensity and volume of exercise, training history, or level of muscle damage. Beyond recovery of performance, which may be arguably the most critical factor regarding recovery strategies, attenuation of soreness and pain may be as important as they can certainly be performance limiters.

The perception of recovery is incommensurate to actual biological repair of damaged muscle cells and therefore an indirect and imperfect marker of EIMD. In other words, the absence of pain or soreness is not directly indicative of complete repair of muscle exposed to EIMD. Nonetheless, the mitigation of DOMS and hyperalgesia remains an important therapeutic concern since the presence of pain at rest or during movement may certainly limit one’s physical performance. Our perception-based markers of EIMD appear to correspond with performance outcomes as presented above. For instance, placebo-treated subjects reported increased rating of soreness under resting conditions up to 48 h post-exercise; however, resveratrol-treated subjects demonstrated elevated soreness compared to baseline only up to 24 h post-exercise. Under muscular tension, both groups demonstrated increased soreness at 24- and 48-h post-exercise when compared to baseline. However, the CTL group exhibited a continual rise between 24 and 48 h while the RES group showed a trend towards baseline levels. When considering the divergent longitudinal trends for soreness between the RES and CTL groups as displayed in Figure 2 and Figure 3, it may be reasonable to suggest that the RES group would experience a faster reduction of soreness under muscular tension than the CTL group. Correspondingly, a propriety resveratrol-based blend investigated by Udani et al. (2009) elicited decreased tenderness 24 h following exercise and lower ratings of soreness at 24 and 48 h post-ECRE when compared to placebo [7]. Also, in a more recent study, Trombold et al. (2011) indicated that supplementation of pomegranate juice, a rich polyphenol source, decreased localized pain in the elbow flexor 2–168 h following a bout of ECRE compared to placebo [5]. The sensation of pain or soreness during EIMD has thought to be partly due to myofibrillar micro tears induced by the initial mechanical stress which in turn triggers an inflammatory response. Additionally, the influx of neutrophils that arise in response to this initial injury stimulate lipid peroxidation and cause a greater rise in free radicals, which are also nociceptive stimuli [3]. Moreover, the innate inflammatory response to EIMD has also been linked to soreness due to the sensitization of muscle nociceptors by various noxious stimuli present during EIMD, e.g., free radicals, inflammatory molecules, and H+ [3]. Because of the connection between pain and oxidative stress and inflammation, resveratrol supplementation may minimize the effect of EIMD on soreness due to potential antioxidant and anti-inflammatory properties [13]. 

Various limitations are inevitable in research studies examining dietary supplements and human performance especially due to inherent physiological/genetic variations among subjects both within and between groups. However, one major control variable widely known to confound data interpretation in such studies is dietary intake. Various methodologies have been used to control for dietary intake especially in cross-sectional investigations; however, most procedures are almost entirely based on subject self-reporting which naturally presents issues with reliability and validity. In the present study, a validated method was utilized for dietary records and no significant differences were found between groups for nutrient and caloric intake. However, no analysis was performed on consumption of organic polyphenols or other bioactive compounds independent of the experimental treatment. This presents a limitation to the current analysis as dietary interference by other bioactive compounds through food consumption remains unknown. Thus, the interpretation of our results involves the assumption that other bioactive compounds from subjects’ food intake were controlled for. Also, the level of resveratrol in vivo was not measured which brings the bioavailability of the test supplement into question. The digestion, absorption, and metabolism of exogenous resveratrol may vary largely among individuals and be influenced by multiple factors, including body mass, enzymatic activity, metabolic rate, and co-consumption of nutrients [13,14]. For example, resveratrol might interact with fatty acids and bind to human plasma lipoproteins during digestion, ultimately reducing absorption [13]. In contrast, protein and nutrients that maintain solubility increase the absorption of resveratrol [13]. 

Overall, the results, at least within the limits of this study, reveal the potential yet minor benefits of a resveratrol-enriched polyphenolic supplement as an exogenous aid for muscle recovery following a bout of damaging exercise. Recreational and perhaps competitive athletes may experience an improved rate of recovery and/or ergogenic response to long-term resveratrol and polyphenol supplementation, especially when recovery time is limited. The results of the current study suggest that the experimental resveratrol-based polyphenol supplement containing a 716 mg blend of polyphenol sources (i.e., muscadine grape extract, red wine, pomegranate, chebulic myrobalan, black currant, purple carrot, and red grape juice extracts) including 60 mg of *trans*-resveratrol/serving may mitigate the decrements to muscular function and performance following a resistance exercise bout. However, considering the vast sources of resveratrol and polyphenols and the variety of bioactive polyphenol compounds, it remains uncertain which specific polyphenols affect mechanisms underlying muscle recovery. Moreover, further research is necessary to identify the optimum dosage in a population-specific manner to better determine supplementation protocols. This remains especially critical since previous animal research shows a dose-dependent response to resveratrol, which does not translate perfectly to human populations [15,28]. Along with using human subjects and examining whole-body outcomes, molecular to cellular-level analyses may further elucidate the vaguely understood mechanisms through which resveratrol and other polyphenols may promote muscle recovery and performance. In conclusion, the present findings demonstrate resveratrol-enriched polyphenol supplementation to be a promising strategy to promote the preservation of performance following a bout of high intensity exercise, although EIMD per se may not be affected.

## Figures and Tables

**Table 1:** Between-group comparison for descriptive measures.

Descriptive Measure	CTL Group (*n* = 12; 6f and 6m)	RES Group (*n* = 10; 6f and 4m)
Age (years)	23.4 ± 2.7	22.3 ± 3.0
TBM (kg)	66.3 ± 8.4	68.3 ± 14.4
LM (kg)	45.0 ± 6.9	46.0 ± 10.3
FM (kg)	18.8 ± 4.4	21.9 ± 7.6
BF% (%)	28.3 ± 5.9	31.2 ± 9.6
BMI (kg/m^2^)	23.4 ± 2.0	25.9 ± 2.8
Height (cm)	170.0 ± 7.4	166.9 ± 7.8

Data presented as mean ± standard deviation. CTL = placebo, RES = resveratrol, TBM = total body mass, LM = lean mass, FM = fat mass, BF% = body fat percentage, BMI = body mass index, f = females, m = males.

**Table 2:** Between-group comparison for pain threshold.

Group	Test Site	Baseline (N)	24 h (N)	48 h (N)
CTL	Vastus intermedius	82.4 ± 27.4	73.4 ± 18.7	74.2 ± 17.5
	Vastus lateralis	71.5 ± 28.4	55.7 ± 14.5 *	59.1 ± 87.8
	Gastrocnemius	86.7 ± 26.0	78.1 ± 24.6	87.7 ± 25.9
RES	Vastus intermedius	71.2 ± 19.2	60.9 ± 23.1	62.3 ± 27.4
	Vastus lateralis	61.6 ± 20.5	55.1 ± 18.8	57.1 ± 15.7
	Gastrocnemius	82.7 ± 34.5	77.4 ± 25.1	72.3 ± 24.2

Data presented as mean ± standard deviation. CTL = placebo, RES = resveratrol; * Significantly different than baseline (*p* = 0.03).

**Table 3:** Between-group comparison for pain tolerance.

Group	Test Site	Baseline (N)	24 h (N)	48 h (N)
CTL	Vastus intermedius	146.1 ± 30.1	140.2 ± 65.3	130.1 ± 31.9 *
	Vastus lateralis	133.1 ± 63.4	99.4 ± 38.6 ^	96.4 ± 37.0 ^
	Gastrocnemius	166.3 ± 55.9	148.0 ± 50.9	141.9 ± 28.3
RES	Vastus intermedius	138.2 ± 52.4	106.5 ± 30.6	110.8 ± 51.3
	Vastus lateralis	98.5 ± 38.1	80.7 ± 23.9	91.8 ± 35.7
	Gastrocnemius	151.4 ± 63.0	130.2 ± 47.5	126.1 ± 52.2

Data presented as mean ± standard deviation. CTL = placebo, RES = resveratrol; * Significantly different than baseline (*p* = 0.03); ^ Significantly different than baseline (*p* = 0.003).
